# Outcomes of extremely preterm infants with neonatal hyperglycemia and hypernatremia: a retrospective cohort study

**DOI:** 10.1038/s41372-026-02683-0

**Published:** 2026-04-13

**Authors:** Anurag Fursule, Gayatri Athalye-Jape, Diksha Deepak, Saravanan Muthusamy, Sanjay Patole, Harshad Panchal, Shripada Rao, Chandra Rath

**Affiliations:** 1 Neonatal Directorate, King Edward Memorial Hospital for Women, Perth, WA Australia; 2 School of Medicine, The University of Western Australia, Perth, WA Australia; 3 Neonatology, Joondalup Health Campus, Joondalup, WA Australia; 4 Paediatrics, Rockingham General Hospital, Rockingham, WA Australia; 5 Neonatal Directorate, Perth Children’s Hospital, Perth, WA Australia; 6 Neonatology, Canberra Hospital, Canberra, ACT Australia

**Keywords:** Risk factors, Neurodevelopmental disorders

## Abstract

**Objective:**

To assess whether early hypernatremia (HN) and/or hyperglycemia (HG) in the first postnatal week are associated with adverse neurodevelopmental outcomes in extremely preterm (EP; <28 weeks) infants.

**Study design:**

This was a single-center retrospective cohort study. HN is defined as serum sodium > 145 mmol/L and HG as blood glucose > 10 mmol/L within days 0–7. Infants were classified into HN, HG, or combined HN + HG groups; EP infants without HN/HG served as controls. This study included 748 EP infants: 374 with HN and/or HG and 374 controls. The primary outcome was an adverse neurodevelopmental outcome at 2 years’ corrected age.

**Results:**

On adjusted analyses, HN, HG and HN + HG were not associated with moderate-to-severe developmental impairment. Moderate-to-severe HN group showed a lower odds of moderate-to-severe developmental impairment than controls (18.3% vs 21.5%; adjusted OR 0.39, 95% CI 0.15–0.99; *p* = 0.047).

**Conclusions:**

Early hypernatremia and/or hyperglycemia were not associated with adverse neurodevelopment at 2 years’ corrected age.

## Introduction

Hypernatremia (HN) and hyperglycemia (HG) are frequently observed in extremely preterm (EP) infants [1, 2]. The incidence of HG varies widely, ranging from 10 to 80%, largely based on the definition of HG [2, 3]. Prematurity is the most important risk factor for HG. The incidence of HN ranges from as low as 2% in term neonates to ~40% in very low birth weight infants, reaching ~70% in infants born at < 27 weeks’ gestation [4–6]. Extremely preterm infants are particularly prone to HN due to high body water content and significant insensible water loss due to a larger surface-to-body weight ratio and immature skin. This, coupled with inefficient water conservation due to renal immaturity, further increases the risk of HN. Regardless of their incidence or underlying aetiologies, both HN and HG are hyperosmolar conditions that can negatively impact organ systems, potentially increasing mortality and morbidity.

A recent systematic review reported a significant association between HG and increased mortality and morbidities such as intraventricular haemorrhage (IVH) and retinopathy of prematurity (ROP) [7]. HG has also been linked to poor cognitive and motor performance at 1 to 6.5 years [8–10]. Reports on the association of HN with mortality and morbidities are limited. However, HN has been linked to an increased likelihood of severe IVH, ROP and lower fine motor and cognitive scores at 18 months [11–14]. HG and HN frequently occur together in the early postnatal life of extremely preterm infants. Considering the role of hyperosmolarity in the pathogenesis of morbidity and mortality, the combination of HN and HG could potentially cause more damage than each condition on its own. Evidence around this hypothesis is scarce. A single-centre retrospective study involving extremely preterm infants has reported an independent association of higher risk of IVH with HN concurrent with HG but not with HN or HG alone on multivariate analyses [15]. Few other studies have explored the association between the duration and severity of HG with adverse outcomes [7].

Given the significance of the underlying issues, we aimed to investigate the association between early HG (>10 mmol/L) and/or HN (>145 mmol/L) with 2-year neurodevelopmental outcome measured using the Bayley scale of infant development in extremely preterm neonates.

## Methods

This was a single-center retrospective cohort study. Approval was obtained from the institution’s quality improvement and audit committee (Reference number: 42525). We used records of all EP infants (gestation < 28 weeks) born in King Edward Memorial Hospital, Perth, between January 2000 and March 2023.

Infants with onset of HN (serum sodium > 145 mmol/L) or HG (blood glucose > 10 mmol/L) within the first 7 days of life were included as cases. Data on neonatal demographic characteristics and outcomes to discharge were collected from the unit’s electronic database. Some of the data, such as peak sodium and glucose levels and duration of HN or HG, were collected from the hospital’s laboratory database. Infants with congenital renal anomalies, metabolic disorders, confirmed meningitis or ventriculitis, and those admitted to our hospital after day 3 of life or transferred to other hospitals, were excluded. The control group infants were selected from the same cohort and matched with cases for gestational age (GA), birth weight (BW) and Clinical Risk Index for Babies (CRIB) score. The case-to-control ratio was 1:1.

The data collection included information on demographic characteristics and outcomes to discharge or death after the first admission. Other variables of interest included GA, BW, gender, growth status, preterm prelabour rupture of membrane (PPROM), antenatal steroids, delivery mode, chorioamnionitis and antepartum haemorrhage (APH). Postnatal variables included APGAR score, CRIB score and outcomes such as IVH, periventricular leukomalacia (PVL), retinopathy of prematurity (ROP), chronic lung disease (CLD), necrotising enterocolitis (NEC), patent ductus arteriosus (PDA), time to full feeds and survival.

All EP infants cared for in our hospital undergo developmental assessment at 2 years corrected age using the Bayley Scales of Infant Development (BSID III or IV). BSID III was introduced in 2009 and BSID IV was introduced in 2019. BSID scores between 70 and 85 (1 SD below mean) were classified as mild delay, while scores < 70 (2 SD below mean) were considered as moderate-severe delay. CLD was defined as the need for oxygen or respiratory support at 36 weeks. Mild HN was defined as a serum sodium > 145 mEq/L but ≤ 150 mEq/L, moderate as > 150 to ≤ 160 mEq/L and severe as > 160 mEq/L. Mild HG was defined as a blood or capillary glucose > 10 mmol/L but ≤ 15 mmol/L and severe as > 15 mmol/L. If the duration of HN or HG was > 24 h but ≤ 48 h, it was classified as prolonged and considered extremely prolonged if it exceeded > 48 h.

The primary aim was to explore the association between HG and/or HN and 2-year neurodevelopmental assessment using Bayley scale of infant development scores.

### Statistical analysis

Continuous data were summarised using median and interquartile range (IQR) or mean and standard deviation (SD) and categorical data were summarised as frequency distributions. Pregnancy, birth and neonatal characteristics and outcomes were compared among HG and HN groups with controls using Kruskal-Wallis tests for continuous outcomes and Chi-square or exact tests for categorical outcomes. Duration of respiratory characteristics was summarised using Kaplan-Meier median, 25^th^ and 75^th^ percentile survival estimates and compared between groups using the Log Rank test. Neurodevelopmental composite scores were analysed using One-Way ANOVA. Disease groups were compared with controls in post hoc pairwise comparisons with the Bonferroni correction for 3 pairwise tests to maintain an overall alpha of 0.05. Neonatal outcomes, including IVH, mortality and neurodevelopmental outcomes were analysed comparing disease groups with controls using unconditional logistic regression modelling. All models were adjusted for GA, CRIB score, NEC and year of birth; mortality was further adjusted for IVH; and neurodevelopmental outcomes for IVH, CLD and corrected age at assessment. Additional analyses were conducted to assess the effect of disease severity and duration on neonatal outcomes. In addition to the previous adjustments, evaluations of HN models were adjusted for severe HG and evaluations of HG models were adjusted for severe HN, as most cases (70%) had both HN and HG. All tests were two-sided and *p*-values < 0.05 were considered statistically significant. IBM SPSS version 29.0 (Armonk, NY) statistical software was used for data analysis.

## Results

A total of 2314 EP infants were admitted to our level 3 NICU between January 2000 and March 2023. Of these, 374 infants who had HN or HG or both were included in the study. Pregnancy and birth characteristics were similar across groups (Table 1). Compared with controls, infants in the combined HG/HN group were more likely to have lower birthweight (*p* < 0.001), Apgar scores < 7 at 1 min (0.017), higher CRIB II scores (*p* < 0.001), require high-frequency jet (*p* = 0.002) and high-frequency oscillatory ventilation (*p *< 0.001), treatment warranting ROP (*p* = 0.029) and CLD (*p* = 0.046). The HN group infants were more likely to have higher GA and BW at birth (both *p* < 0.001), lower CRIB II scores (*p* < 0.001), short duration of ventilation (*p* < 0.001) and time to reach full feeds (*p* = 0.006) compared to controls. The demographic characteristics were not significantly different between the HG and control group infants (Table 1).

**Table 1:** Pregnancy and neonatal characteristics.

	Control *N* = 374	Hyperglycaemia *N* = 68	Hypernatremia *N* = 43	Both *N* = 263	*p*-value
	*N* (%)	*N* (%)	*N* (%)	*N* (%)	
*Pregnancy & birth characteristics*					
Antenatal steroid complete	166 (44.4)	33 (48.5)	14 (32.6)	134 (51.0)	0.101
IUGR	66 (17.6)	17 (25.0)	5 (11.6)	47 (17.9)	0.325
Amnionitis	64 (17.1)	10 (14.7)	7 (16.3)	46 (17.5)	0.957
APH	130 (34.8)	21 (30.9)	18 (41.9)	98 (37.3)	0.610
PPROM	111 (29.7)	15 (22.1)	17 (39.5)	95 (36.1)	0.066
Inborn	350 (93.6)	67 (98.5)	39 (90.7)	243 (92.4)	0.274
CS delivery	197/373 (52.8)	43 (63.2)	17/42 (40.5)	123 (46.8)	0.040
*Neonatal characteristics*					
Gestational age (w) (median, IQR)	25 (24–26)	25 (24–26)	26 (24–27)^a^	24 (24–25)	<0.001
Birthweight (g) (median, IQR)	705 (605–839)	708 (599–804)	815 (715–975)^b^	680 (570–790)^a^	<0.001
Male	202 (54.0)	34 (50.0)	21 (48.8)	153 (58.2)	0.466
Apgar < 7 at 1 min	295 (79.3)	54 (79.4)	32 (78.0)	231 (88.5)^a^	0.017
Apgar < 7 at 5 min	126 (33.9)	20 (29.4)	13 (31.7)	97 (37.3)	0.595
CRIB II score^#^	14 (12–15)	13 (11–14)	12 (10–14)^b^	14 (13–16)^a^	<0.001
Intubation/IPPV	33 (8.8)	8 (11.8)	3 (7.0)	23 (8.7)	0.838
Ventilation (h) (median, IQR)	419 (87–886) *N* = 358	419 (67–764) *N* = 67	89 (23–593)^a^ *N* = 40	673 (240–990) *N* = 256	<0.001
HFJV	52 (13.9)	13 (19.1)	8 (18.6)	69 (26.2)^a^	0.002
HFOV	82 (21.9)	12 (17.6)	5 (11.6)	91 (34.6)^a^	<0.001
CPAP (h) (median, IQR)	1079 (790–1338) *N* = 307	1196 (930–1392) *N* = 60	1037 (791–1297) *N* = 37	1086 (833–1303) *N* = 208	0.607
Oxygen (h) (median, IQR)	1595 (815–2468) *N* = 366	1501 (804–2589) *N* = 65	1166 (305–1854) *N* = 40	1818 (1062–2713) *N* = 258	0.016
Postnatal steroids	64 (17.1)	15 (22.1)	5 (11.6)	65 (24.7)	0.053
Vasopressors	48 (12.8)	12 (17.6)	5 (11.6)	49 (18.6)	0.191
*Neonatal outcomes*					
Sepsis	125 (33.4)	19 (28)	11 (25.6)	77(29.5)	
EOS	9 (7.2)	2 (10.5)	1 (9.1)	7 (9.1)	
LOS	116 (92.8)	23 (89.5)	10 (90.9)	83 (90.9)	
ROP requiring treatment	56 (15.0)	5 (7.4)	2 (4.7)	48 (18.3)	0.029
NEC Stage 2/3	9 (2.4)	3 (4.4)	1 (2.3)	15 (5.7)	0.163
PDA requiring treatment	236 (63.1)	47 (69.1)	22 (51.2)	179 (68.1)	0.124
IVH Grade 3/4	54 (14.4)	9 (13.2)	4 (9.3)	47 (17.9)	0.395
PVL	7 (1.9)	1 (1.4)	2 (4.5)	12 (4.6)	0.176
CLD	232 (62.0)	48 (70.6)	22 (51.2)	182 (69.2)	0.046
Age at full feeds (d) (median, IQR)	13 (10–20) *N* = 299	15 (10–20) *N* = 55	9 (7–15)^a^ *N* = 37	14 (9–21) *N* = 209	0.006
Suck feed at discharge	235/294 (79.9)	44/55 (80.0)	32/37 (86.5)	164/204 (79.6)	0.806
Died before discharge^##^	55/349 (15.8)	9 (13.2)	4 (9.3)	44 (16.7)	0.604

*CRIB* Clinical Risk Index for Babies, *IUGR* intrauterine growth restriction, *APH* antepartum haemorrhage, *PPROM* preterm premature rupture of membranes, *CS* caesarean section, *IQR* interquartile range, HFJV high-frequency jet ventilation, *HFOV* high-frequency oscillatory ventilation, *CPAP* continuous positive airway pressure, *EOS* early onset sepsis, LOS late onset sepsis, *ROP* retinopathy of prematurity, *NEC* necrotising enterocolitis, *PDA* patent ductus arteriosus, *IVH* intraventricular haemorrhage, *PVL* periventricular leukomalacia, *CLD* Chronic lung disease.

^a^*p* < 0.05 compared to control.

^b^*p* < 0.01 compared to control.

^#^CRIB score missing (*n* = 7).

^##^ ‘Unknown’ outcome at discharge (*n* = 24).

There were no significant differences in neurodevelopmental outcomes between cases and controls in univariate analyses (Table 2). There was also an overall difference in the mean composite language scores between groups in Table 2 (*p*-value of 0.023); however, there were no differences between control, HN, HG or those with both conditions groups in adjusted pairwise comparisons at the *p* < 0.05 level (HG vs both conditions, *p* = 0.089 with Bonferroni adjustment). There was a statistically significant difference in language delay (<85) between the ‘HG’ (23.7%) and ‘both HN and HG’ groups (48.4%); overall *p* = 0.017, but not between the control and any of the disease groups.

**Table 2:** Neurodevelopmental outcomes.

	Control	Hyperglycaemia	Hypernatremia	Both	*p*-value (Overall)
	*N* = 154	*N* = 41	*N* = 23	*N* = 143	
Corrected age at assessment (m) (median, IQR)	24 (24–25)	24 (24–25)	24 (24–25)	24 (24–25)	0.277
Cognitive	*N* = 148	*N* = 41	*N* = 21	*N* = 140	
Composite (mean, SD)	96.1 (16.9)	98.7 (16.8)	101.0 (12.0)	94.6 (16.3)	0.257
<85 (*n*, %)	31 (20.9)	5 (12.2)	1 (4.8)	23 (16.4)	0.214
<70 (*n*, %)	9 (6.1)	2 (4.9)	1 (4.8)	13 (9.3)	0.700
Language	*N* = 137	*N* = 38	*N* = 19	*N* = 128	
Composite (mean, SD)	89.2 (19.8)	94.0 (19.0)	95.6 (16.3)	85.0 (20.1)	0.023^a^
<85 (*n*, %)	50 (36.5)	9 (23.7)	5 (26.3)	62 (48.4)	0.017
<70 (*n*, %)	22 (16.1)	3 (7.9)	1 (5.3)	28 (21.9)	0.092
Motor	*N* = 139	*N* = 37	*N* = 20	*N* = 137	
Composite (mean, SD)	92.6 (17.2)	94.1 (18.0)	97.0 (12.3)	93.2 (15.9)	0.731
<85 (*n*, %)	33 (23.7)	6 (16.2)	3 (15.0)	28 (20.4)	0.662
<70 (*n*, %)	12 (8.6)	4 (10.8)	0 (0)	12 (8.8)	0.548
Social-emotional	*N* = 138	*N* = 40	*N* = 20	*N* = 135	
Composite (mean, SD)	99.2 (16.5)	101.0 (18.2)	106.5 (16.2)	97.7 (18.0)	0.174
<85 (*n*, %)	19 (13.8)	5 (12.5)	2 (10.0)	26 (19.3)	0.478
<70 (*n*, %)	9 (6.5)	2 (5.0)	0 (0)	9 (6.7)	0.697

*SD* Standard deviation, *IQR* Interquartile range, *m* Months.

^a^Bonferroni-adjusted pairwise comparisons were not statistically significant between groups.

### Neonatal outcomes (Adjusted analysis)

There were no associations observed between HG/HN groups and the control group for IVH Grade 3/4, mortality and/or moderate to severe developmental delay (composite scores < 70) (Table 3). Disease groups were collapsed into one group to provide sufficient numbers for analysis by individual scale. No associations were observed between cases and controls for moderate to severe cognitive, language, motor or social-emotional delay (Table 3).

**Table 3:** Neonatal and neurodevelopmental outcomes (Adjusted analyses).

	Unadjusted OR (95% CI)	*p*-value	Adjusted OR (95% CI)	*p*-value
*Outcome*				
IVH Grade ¾^#^				
Control	1.00		1.00	
Hyperglycaemia	0.92 (0.43–1.97)	0.835	1.20 (0.54–2.66)	0.653
Hypernatremia	0.66 (0.23–1.93)	0.449	0.88 (0.29–2.71)	0.823
Both	1.31 (0.85–2.02)	0.223	1.06 (0.66–1.68)	0.822
Mortality^##^				
Control	1.00		1.00	
Hyperglycaemia	0.84 (0.39–1.80)	0.657	1.06 (0.46–2.44)	0.894
Hypernatremia	0.44 (0.13–1.48)	0.184	0.66 (0.17–2.52)	0.466
Both	1.13 (0.73–1.75)	0.578	0.88 (0.53–1.47)	0.631
*Neurodevelopmental assessment*^###^				
Moderate-severe delay (any)				
Control	1.00		1.00	
Hyperglycaemia	0.40 (0.13–1.19)	0.099	0.36 (0.11–1.14)	0.082
Hypernatremia	0.18 (0.02–1.42)	0.104	0.19 (0.02–1.51)	0.117
Both	1.14 (0.66–1.98)	0.646	1.02 (0.57–1.85)	0.946
Mortality/Mod-severe delay (any)				
Control	1.00		1.00	
Hyperglycaemia	0.40 (0.13–1.21)	0.104	0.37 (0.12–1.17)	0.090
Hypernatremia	0.17 (0.02–1.34)	0.094	0.18 (0.02–1.44)	0.106
Both	1.17 (0.67–2.02)	0.587	1.03 (0.57–1.84)	0.932
Mortality/Moderate-severe cognitive delay				
Control	1.00		1.00	
Hyperglycaemia	0.81 (0.17–3.88)	0.787	0.90 (0.17–4.78)	0.906
Hypernatremia	0.73 (0.09–6.07)	0.773	0.78 (0.09–7.17)	0.828
Both	1.64 (0.68–3.96)	0.274	1.44 (0.55–3.73)	0.458
Moderate-severe cognitive delay				
Control	1.00		1.00	
Cases	1.34 (0.57–3.11)	0.502	1.26 (0.51–3.15)	0.619
Moderate-severe language delay				
Control	1.00		1.00	
Cases	1.09 (0.60–1.98)	0.769	0.98 (0.53–1.83)	0.960
Moderate-severe motor delay				
Control	1.00		1.00	
Cases	0.96 (0.44–2.09)	0.912	0.89 (0.38–2.10)	0.795
Moderate-severe social-emotional delay				
Control	1.00		1.00	
Cases	0.86 (0.35–2.14)	0.748	0.82 (0.31–2.15)	0.683

^#^ adjusted for GA, CRIB score, NEC and year of birth.

^##^ adjusted for GA, CRIB score, NEC, IVH and year of birth.

^###^ adjusted for GA, CRIB score, NEC, IVH, CLD, year of birth and corrected age at assessment.

*OR* odds ratio, *CI* confidence interval, *GA* Gestational age, *CRIB* Clinical Risk Index for babies, *NEC* necrotising enterocolitis, *IVH* intraventricular haemorrhage, *CLD* Chronic lung disease.

### Disease severity, duration and outcomes

Tables 4 and 5 present the relationship between disease severity and duration and neonatal outcomes. Severe HN was associated with IVH Grade 3/4 compared with the control group (70.6% vs 14.4%, adjusted OR 11.4, 95% CI 3.3–39.3, *p* < 0.001). It was not significantly associated with mortality, except when the adjustment for IVH was removed from the model (OR 4.3, 95% CI 1.4–13.3, *p* = 0.010), suggesting the effect of severe HN on increased mortality may be mediated by the association with IVH. There was a reduction in overall moderate to severe delay (in any developmental domain) in the moderate to severe HN group compared with the controls (18.3% vs 21.5%, aOR 0.39, 95% CI 0.15–0.99, *p* = 0.047) in adjusted analysis. No associations were observed between severity or duration of HG and IVH, mortality or neurodevelopmental outcomes.

**Table 4:** Neonatal outcomes and association with disease severity and duration.

Outcome	*N* (%)	Unadjusted OR (95% CI)	Adjusted OR (95% CI)	*p*-value
IVH Grade 3/4				
Hypernatremia				
Control	54/374 (14.4)	1.00	1.00	
Mild	16/164 (9.8)	0.62 (0.34–1.13)	0.56 (0.29–1.09)	0.088
Moderate	23/123 (18.7)	1.40 (0.82–2.40)	0.91 (0.48–1.73)	0.781
Severe	12/17 (70.6)	18.06 (5.61–58.08)	11.44 (3.33–39.33)	<0.001
Duration of hypernatremia				
Control	54/374 (14.4)	1.00	1.00	
≤24 h	21/107 (19.6)	1.42 (0.80–2.50)	1.06 (0.56–1.99)	0.865
>24 to ≤48 h	13/93 (14.0)	0.99 (0.52–1.91)	0.83 (0.41–1.68)	0.601
>48 h	17/105 (16.2)	1.19 (0.66–2.16)	0.90 (0.45–1.80)	0.763
Hyperglycaemia				
Control	54/374 (14.4)	1.00	1.00	
Mild	39/251 (15.5)	1.09 (0.69–1.71)	0.83 (0.50–1.38)	0.470
Severe	17/80 (21.3)	1.65 (0.90–3.04)	1.38 (0.70–2.71)	0.350
Duration of hyperglycaemia				
Control	54/374 (14.4)	1.00	1.00	
≤24 h	30/143 (21.0)	1.56 (0.94–2.57)	1.18 (0.67–2.06)	0.575
>24 to ≤48 h	11/78 (14.1)	0.99 (0.49–1.99)	0.76 (0.35–1.63)	0.477
>48 h	15/110 (13.6)	0.97 (0.52–1.80)	0.81 (0.41–1.61)	0.555
Mortality				
Hypernatremia				
Control	55/350 (15.7)	1.00	1.00	
Mild	13/164 (7.9)	0.48 (0.25–0.90)	0.52 (0.26–1.07)	0.074
Moderate	25/123 (20.3)	1.40 (0.83–2.38)	1.18 (0.61–2.28)	0.627
Severe	10/17 (58.8)	7.00 (2.50–19.60)	2.37 (0.73–7.75)	0.153
Duration of hypernatremia				
Control	55/350 (15.7)	1.00	1.00	
≤24 h	21/107 (19.6)	1.36 (0.78–2.38)	1.04 (0.53–2.03)	0.907
>24 to ≤48 h	14/93 (15.1)	0.98 (0.52–1.85)	0.95 (0.46–1.96)	0.886
>48 h	13/105 (12.4)	0.72 (0.37–1.40)	0.58 (0.26–1.32)	0.193
Hyperglycaemia				
Control	55/350 (15.7)	1.00	1.00	
Mild	38/251 (15.1)	0.99 (0.63–1.55)	0.80 (0.47–1.35)	0.396
Severe	15/80 (18.8)	1.28 (0.68–2.40)	1.01 (0.48–2.11)	0.977
Duration of hyperglycaemia				
Control	55/350 (15.7)	1.00	1.00	
≤24 h	26/143 (18.2)	1.23 (0.74–2.06)	0.88 (0.48–1.61)	0.670
>24 to ≤48 h	15/78 (19.2)	1.30 (0.69–2.44)	1.02 (0.49–2.11)	0.964
>48 h	12/110 (10.9)	0.68 (0.35–1.33)	0.67 (0.31–1.42)	0.292

Adjusted for GA, CRIB score, NEC, year of birth (all outcomes) and IVH (mortality outcome only).

*OR* odds ratio, *CI* confidence interval, *GA* Gestational age, *CRIB* Clinical Risk Index for babies, *NEC* necrotising enterocolitis, *IVH* intraventricular haemorrhage.

**Table 5:** Neurodevelopmental outcomes and association with disease severity and duration.

	N (%)	Unadjusted OR (95% CI)	Adjusted OR (95% CI)	*p*-value
Mod-severe delay (any)				
Hypernatremia				
Control	32/149 (21.5)	1.00	1.00	
Mild	22/100 (22.0)	1.03 (0.56–1.91)	0.78 (0.38–1.59)	0.490
Moderate-severe	11/60 (18.3)	0.84 (0.39–1.80)	0.39 (0.15–0.99)	0.047
Duration of hypernatremia				
Control	32/149 (21.5)	1.00	1.00	
≤24 h	14/57 (24.6)	1.19 (0.58–2.44)	0.79 (0.35–1.80)	0.577
>24 to ≤48 h	9/46 (19.6)	0.89 (0.39–2.03)	0.62 (0.24–1.60)	0.327
>48 h	10/57 (17.5)	0.80 (0.36–1.75)	0.44 (0.17–1.11)	0.082
Hyperglycaemia				
Control	32/149 (21.5)	1.00	1.00	
Mild	24/147 (16.3)	0.72 (0.40–1.29)	0.70 (0.38–1.30)	0.262
Severe	13/34 (38.2)	2.26 (1.02–5.01)	1.80 (0.75–4.31)	0.189
Duration of hyperglycaemia				
Control	32/149 (21.5)	1.00	1.00	
≤24 h	13/78 (16.7)	0.73 (0.36–1.49)	0.77 (0.36–1.63)	0.491
>24 to ≤48 h	8/43 (18.6)	0.84 (0.35–1.98)	0.89 (0.36–2.22)	0.810
>48 h	16/60 (26.7)	1.36 (0.68–2.73)	0.98 (0.46–2.08)	0.948
Mortality/mod-severe delay (any)				
Hypernatremia				
Control	87/355 (24.5)	1.00	1.00	
Mild	35/164 (21.3)	0.86 (0.55–1.35)	0.76 (0.45–1.27)	0.291
Moderate	35/123 (28.5)	1.25 (0.79–1.98)	0.80 (0.46–1.41)	0.443
Severe	11/17 (64.7)	5.17 (1.83–14.65)	1.52 (0.46–4.99)	0.489
Duration of hypernatremia				
Control	87/355 (24.5)	1.00	1.00	
≤24 h	35/107 (32.7)	1.55 (0.97–2.49)	1.04 (0.60–1.81)	0.880
>24 to ≤48 h	23/93 (24.7)	1.04 (0.61–1.76)	0.83 (0.46–1.51)	0.538
>48 h	23/105 (21.9)	0.84 (0.50–1.43)	0.55 (0.29–1.04)	0.067
Hyperglycaemia				
Control	87/355 (24.5)	1.00	1.00	
Mild	62/251 (24.7)	1.04 (0.71–1.51)	0.85 (0.56–1.30)	0.452
Severe	28/80 (35.0)	1.71 (1.01–2.87)	1.30 (0.73–2.33)	0.374
Duration of hyperglycaemia				
Control	87/355 (24.5)	1.00	1.00	
≤24 h	39/143 (27.3)	1.19 (0.76–1.85)	0.93 (0.57–1.52)	0.763
>24 to ≤48 h	23/78 (29.5)	1.30 (0.75–2.24)	0.95 (0.51–1.77)	0.865
>48 h	28/110 (25.5)	1.09 (0.66–1.78)	0.75 (0.40–1.42)	0.376

Adjusted for GA, CRIB score, IVH, NEC, CLD, year of birth and corrected age at assessment.

*OR* odds ratio, *CI* confidence interval, *h* hour, *GA* Gestational age, *CRIB* Clinical Risk Index for babies, *NEC* necrotising enterocolitis, *IVH* intraventricular haemorrhage.

## Discussion

Our study shows that extremely preterm neonates with both HN and HG tend to be significantly smaller, more critically ill and born at a lower gestational age. While this combination may be associated with short-term complications such as IVH, CLD, and ROP requiring treatment, it does not appear to be associated with major adverse long-term outcomes.

It has been hypothesized that adverse neurodevelopmental outcomes in preterm neonates exposed to hyperosmolarity may be linked to neuronal cell damage and IVH early in life. Additionally, the association between HG or HN and short-term complications such as ROP and CLD could contribute to adverse long-term outcomes. In this context, it is crucial to explore the pathophysiological mechanisms underlying these outcomes, considering the findings from our study.

Unlike in paediatric and adult populations, newborns, particularly EP neonates, are highly susceptible to plasma osmotic disturbances caused by HG and HN. We anticipated adverse outcomes in the HG/HN group. However, our findings revealed a high incidence of IVH only in the severe HN group. These unexpected findings may be better understood through insights gained from animal models [16–18]. When plasma osmolality increases, water flows across the blood-brain barrier down its concentration gradient from brain to plasma, and brain volume decreases. The brain responds to this stress by gaining osmotically active solutes, which limit water loss. This phenomenon is termed brain volume regulation. During acute hyperosmolality, the adult sheep cortex loses more water than the rat brain, about three-fourths versus one-third of that predicted by ideal osmotic behaviour indicating a species-specific difference in volume regulation [17, 18]. It is possible that the preterm human brain may have more effective volume regulation, making it more resistant to wide osmotic changes in plasma. The development of brain volume regulation in preterm lambs is known to be gestation-dependent and occurs in stages, beginning in the cerebral cortex and cerebellum, followed later by the medulla [18]. Differences in regional maturation of the brain may explain findings of domain-specific rather than global developmental delay.

Hypertonic plasma draws water from the brain parenchyma, causing tissue shrinkage, which can stretch blood vessels and potentially result in IVH. However, IVH is multifactorial and often occurs in the absence of plasma hyperosmolarity. Mechanisms such as Donnan exclusion, neurons coated with a proteoglycan matrix, reduced functional aquaporin channels and stable NKCC1-expressing preterm neurons can limit water exchange [16]. The neurons and cerebral blood vessels likely tolerate variations in plasma osmolarity to some extent. Nevertheless, hypoxic-ischemic stress and cell membrane damage may overwhelm these protective mechanisms, leading to cell shrinkage and subsequent IVH [16]. In our study, only severe hypernatremia (>160 mmol/L) and not hyperglycemia, was associated with a significantly increased incidence of grade 3/4 IVH. If IVH is truly related to osmolarity, the high incidence observed exclusively in the severe HN group—rather than in the combined HN and HG group—remains unexplained. It is possible that rapid fluctuations in sodium levels, the greater impact of sodium compared to glucose on osmolarity [19], or overly aggressive correction of HN contributed to this finding [20].

Our study revealed an unexpected protective effect of moderate to severe HN on neurodevelopmental delay, showing an association in the opposite direction of what was hypothesized. This finding may be influenced by survivor bias, as only the infants who survived were available for neurodevelopmental assessment. Additionally, the low follow-up rate in this subset of neonates and the fact that the p-value is at the threshold of statistical significance suggest that larger studies are needed to validate this effect.

To the best of our knowledge, only one other case-control study has reported an association between HN, HG and IVH in adjusted analyses. However, it did not identify any association between hyperglycaemia or hypernatremia alone and IVH [15]. The low baseline incidence of HN or HG in our cohort and differences in the definitions of HN or HG may explain such differences in results. It is important to note that, except for a significant association between severe HN and IVH, subgroup analyses of graded or persistent HN and HG in our study did not show any significant results.

Our results suggest the need for large, well-designed prospective cohort studies reporting adjusted odds or risk ratios to generate robust evidence in the area. Analyses adjusting for important confounding factors are crucial when evaluating long-term neurodevelopmental outcomes. Evidence from follow-up studies conducted at pre-school age would be more reliable, considering the difficulties in assessing cognition at early ages. Unfortunately, such data are limited, making it challenging to draw definitive conclusions. Existing studies examining the association between HG or HN and developmental outcomes have yielded conflicting results, partly due to variations in the cutoff values used to define HG and HN across different cohorts [7, 12, 14, 21]. Assuring uniformity in the definitions of HG or HN and standardization of long-term developmental assessments is critical to minimise the risk of variation in results due to differences in methodology. Limited evidence suggests that insulin treatment does not improve short-term or neurodevelopmental outcomes in infants with hyperglycaemia [22]. This raises an important question about how aggressively we should manage these osmotic imbalances in clinical practice in the context of our results.

The strengths of our study include the use of matched controls, long-term follow-up data, the largest sample size to date compared to previous studies, and robust statistical analyses. However, being a retrospective analysis from a single centre, the external validity of our findings is limited. Two versions of the BSID were used during the study period, which may introduce variability in outcome assessment. The lack of adjustment for post-discharge factors such as parental education, socioeconomic status and home environment may have influenced our results. Additionally, we cannot rule out the potential impact of unknown confounders or changes in clinical practices over the course of the study period.

## Conclusion

Our results show that exposure to HN and HG in early postnatal life may not be associated with adverse long-term outcomes. Large, well-designed prospective cohort studies are required to confirm our findings.

## Data Availability

The datasets generated during and/or analysed during the current study are available from the corresponding author on reasonable request.
